# Constructing preschool children’s music subject key competencies in Henan province, China: A theoretical study based on interviews with kindergarten teachers and experts

**DOI:** 10.1371/journal.pone.0348052

**Published:** 2026-11-13

**Authors:** Yanan Lu, Lin Shi, Ahmad Faudzi Musib

**Affiliations:** 1 Center for Post-doctoral Studies of Sport Science, Chengdu Sport University, Chengdu, Sichuan, China; 2 School of Physical Education, Chengdu Sport University, Chengdu, Sichuan, China; 3 Department of Music, Faculty of Human Ecology, Universiti Putra Malaysia, Serdang, Selangor, Malaysia; East China Normal University, CHINA

## Abstract

**Background:**

Competency-based reforms increasingly position preschool music as a foundation for children’s holistic development. Yet in China, an operational and locally grounded framework of music subject key competencies for preschool children aged 3–6 remains under-specified, limiting the alignment of curriculum goals, classroom activities, and process-oriented assessment.

**Methods:**

This study used an inductive qualitative design. Purposive stratified sampling and snowball sampling were used to recruit 30 participants (kindergarten teachers, managers, and preschool/music education experts) from model kindergartens and universities in Henan Province. Individual semi-structured interviews were conducted. Data were managed in NVivo 12 Plus and analyzed using an inductive qualitative approach informed by grounded theory coding procedures.

**Results:**

The analysis yielded a seven-dimension framework of music subject key competencies—Music Aesthetic Perception, Artistic Expression, Musical Social Skills, Cultural Understanding, Autonomous Development, Imagination and Creativity, and Physical Movement—together with 24 secondary indicators and observable behaviours. The framework conceptualises early music learning as an integrated developmental process linking perception, embodied action, creative expression, social participation, and cultural meaning-making.

**Conclusions:**

The framework offers music-centered, practice-grounded guidance for preschool music curriculum design and process-focused assessment, and provides a foundation for subsequent scale development, observation tools, and intervention studies in early childhood music education.

## Introduction

In recent years, global basic education has shifted from knowledge-based learning toward competency-oriented curriculum and assessment reform. The concept of “key competencies” was first systematically articulated in international education discourse by the OECD DeSeCo project, which emphasized individuals’ capacity to mobilize knowledge, skills, attitudes, and values to respond to complex demands in specific contexts [1]. The European Commission later defined key competences for lifelong learning as those necessary for personal fulfilment, social inclusion, active citizenship, and employability in a knowledge society [2]. In the Chinese educational context, key competencies are intended to foster the essential character traits and key abilities required for lifelong development and social development. This concept integrates knowledge, skills, emotions, attitudes, values, and other developmental attributes [3]. Applied to preschool music education, this perspective provides a broader conceptual lens for understanding children’s musical learning beyond isolated skill acquisition.

Accordingly, this study adopts a competency-based perspective to examine music subject key competencies in preschool education. Although key competencies provide a general orientation for educational development, their realization requires interpretation within specific subject domains. In music education, the development of subject-specific competencies requires attention to the distinctive ways in which children engage with music. Previous research has emphasized that music subject key competencies are not equivalent to musical techniques or professional performance abilities; rather, they reflect the unique educational value of music and its contribution to learners’ overall development within curriculum contexts [4]. From this perspective, preschool music subject key competencies refer to the integrated capacities that children develop through musical experiences, reflecting both the characteristics of musical learning and the developmental needs of young children.

In China, subject key competencies have been more clearly articulated in compulsory education and related curriculum reforms. The Compulsory Education Arts Curriculum Standards (2022 Edition) identify four core competencies—Aesthetic Perception, Artistic Expression, Creative Practice, and Cultural Understanding [5]. However, these competencies are formulated for compulsory education and do not specify how they should be interpreted and organized for children aged 3–6. At the preschool level, however, the articulation of key competencies at the level of specific subject areas remains less explicit, particularly in music education. The Guidelines for Learning and Development of Children Aged 3–6 Years [6] issued by the Ministry of Education of the People’s Republic of China, constitute a national-level framework that provides a common reference for preschool education across China’s provinces, autonomous regions, and municipalities. Within the arts domain, the Guidelines set out broad developmental aims mainly through two areas: “Perception and Appreciation” and “Expression and Creation.” Children are encouraged to develop interest in beauty in nature and daily life, appreciate diverse forms of artistic expression and works, participate willingly in artistic activities, express themselves confidently, and develop preliminary capacities for artistic expression and creation. Although local education authorities and kindergartens may adapt implementation through local curriculum resources and kindergarten-based practices, music remains embedded within the broader arts domain and is not further specified in terms of preschool children’s music subject key competencies. Nor does the framework provide detailed operational guidance for translating these general arts aims into music curriculum planning, classroom implementation, or systematic assessment [7–9].

A similar issue can also be observed in other Asian preschool curriculum frameworks. In Malaysia’s National Preschool Standard Curriculum (Kurikulum Standard Prasekolah Kebangsaan), music is included within the Creativity and Aesthetics strand [10]. Similarly, Singapore’s Nurturing Early Learners Framework positions music, movement, visual arts, and drama within the learning area of Aesthetics and Creative Expression [11]. These frameworks include elements such as listening, rhythmic response, creative movement, sound exploration, imagination, and collaborative participation [10,11]. However, music remains organized within broader aesthetic and creative learning domains rather than as an independently articulated system of music subject key competencies. Moreover, the competency emphases and organizational structures of these frameworks are embedded within their own national curriculum contexts and therefore cannot directly determine what should constitute music subject key competencies for children aged 3–6 in China. Thus, although these frameworks provide important conceptual and curricular references, they leave unresolved how music-specific, developmental, and broader learning competencies should be integrated and organized within the Chinese preschool context.

Previous research on early childhood curriculum reform in China has documented discrepancies between intended curriculum goals and their interpretation and enactment in practice. In particular, teachers may have difficulty distinguishing among broader educational goals, curriculum goals, and specific teaching goals, and differences have been reported between policy intentions and classroom implementation [12,13]. When policy documents or curriculum remain at the level of broad developmental aims, teachers may find it difficult to translate these aims into coherent classroom goals, activities, and evidence of learning. This challenge is particularly evident in preschool education, where curriculum standards and teaching materials are less unified than in compulsory education, and local practices may vary considerably [14–16]. In addition, increased curriculum autonomy may create both opportunities and risks. While it allows kindergartens to adapt activities to children’s interests and local resources, it may also lead to “schoolification,” excessive emphasis on knowledge transmission, and reduced attention to play-based and inquiry-based experiences [17–19].

This issue is especially relevant to preschool music education. Existing studies suggest that music activities may support children’s emotional growth, social-emotional competence, and self-regulation [20–23]. Empirical studies indicate that some preschool music practices show skills-oriented and reproductive tendencies, including repeated rhythmic practice, emphasis on accuracy, and more frequent reproductive than creative activities [24,25]. Thus, the concern is not technical learning itself, but whether it is balanced with play-based exploration, aesthetic experience, improvisation, collaboration, and children’s autonomous expression [24–27]. At the assessment level, although policy documents advocate an evaluation system oriented toward children’s holistic development, frontline practice still frequently relies on outcome-focused checks with narrow and largely subjective criteria. When observation is unplanned and documentation lacks analytic follow-through, insufficient process evidence constrains teachers’ capacity to identify, feed back, and accumulate learning gains [15,16,28,29]. Importantly, greater clarity in articulating preschool music competencies should not be equated with more outcome-oriented assessment. Developmentally appropriate assessment emphasizes ongoing observation and interpretation of children’s learning in context, rather than judgment against fixed performance thresholds [30,31]. From this perspective, a music subject key competencies framework may serve as a pedagogical reference for understanding children’s evolving musical learning and informing curriculum and formative observation, rather than as a checklist of outcomes to be mastered.

Although preschool music education is widely recognized as important for children’s development, there remains limited clarity regarding what constitutes music subject key competencies for children aged 3–6, how these competencies relate to one another, and how they can be organized into a developmentally appropriate framework that reflects the characteristics of preschool children’s music learning within the Chinese educational context. To address this issue, the present study used semi-structured interviews with kindergarten teachers, managers, and experts in preschool education and music education to explore and synthesize their conceptualizations of music subject key competencies for children aged 3–6 and, on this basis, develop a preliminary, practice-grounded conceptual framework within the Chinese preschool context. Specifically, this study addressed the following research question: How do kindergarten-based practitioners and experts in preschool and music education conceptualize the key dimensions and indicators of music subject key competencies for children aged 3–6? Using an inductive qualitative analysis, this study aimed to identify the key dimensions and indicators through which preschool children’s music subject key competencies can be conceptualized. The resulting framework may provide an empirical basis for future curriculum development, classroom observation, and formative assessment research in preschool music education.

## Research methods

### Study setting

Henan Province, China, was selected as the research site because it is a populous central province with a large preschool sector and a well-established provincial model kindergarten system, enabling access to information-rich practice-based cases [32]. To capture within-province variation, a stratified site-selection strategy was adopted, and three administrative levels were covered: Zhengzhou (provincial capital), Pingdingshan (prefecture-level city), and Wugang (county-level city), reflecting differences in regional development and educational resources [33].

At the kindergarten level, only institutions with over 20 years of operation, dedicated music classrooms, and regular music courses and performances were included. Final selection prioritized kindergartens with sound management, high educational quality, and strong cooperation willingness.

### Sampling of interview participants

This study used a combination of purposive stratified sampling and snowball sampling. In the first stage, purposive stratified sampling was used to identify information-rich participants from different geographical areas, kindergarten types, and classroom levels. The strata included geographical location (provincial capital, prefecture-level city, and county-level city), kindergarten type (mainly public model kindergartens, with several selected high-quality private kindergartens), and class level (senior, middle, and junior preschool classes). Model kindergartens were initially selected because they had relatively stable institutional histories, experience in music-related teaching or performance activities, and more systematic engagement with preschool curriculum practice. These criteria were used to support the construction of an expert- and practice-informed framework, rather than to produce a statistically representative sample of all Chinese kindergartens.

In the second stage, snowball sampling was used within the predefined participant categories. Initial participants and professional contacts were asked to recommend additional teachers, managers, or experts who had relevant experience in preschool music education, curriculum development, teacher training, or preschool education research. Participants were included if they had been continuously engaged in preschool music teaching, research, or management for at least three years and agreed to the recording and anonymized use of the interview data.

Thirty participants were ultimately included: 25 kindergarten-based practitioners and 5 experts from universities or research institutes specializing in preschool or music education. The kindergarten-based practitioners included both teachers and managers. They were grouped together because the managers had developed from frontline teaching positions and remained closely involved in teaching, curriculum planning, teaching research, or classroom guidance. Therefore, they were regarded as practice-based participants within kindergarten settings. The sample represented a range of ages, teaching experience, and educational backgrounds, enabling a multidimensional understanding of preschool children’s music subject key competencies. Participants’ basic information is presented in Table 1.

**Table 1:** Demographic information of interview participants (N = 30).

Participant group	N	Gender	Age range (years)	Teaching/working experience (years)	Highest qualification
Kindergarten teachers/managers	25	All Female	25–45 (≈ M 31)	3–23 (≈ M 9)	6 Associate, 17 Bachelor, 2 Master
University experts	5	4 Female, 1 Male	42–80 (≈ M 52)	19–54 (≈ M 28)	3 Master, 2 Doctor

Note. Kindergarten-based practitioners include kindergarten teachers and managers.

As shown in Table 1, all kindergarten-based practitioners were female. This reflects the gender structure of the preschool education workforce in China, where kindergarten teachers are predominantly female and male teachers are relatively rare, especially in daily music teaching. Therefore, the practitioner perspectives captured in this study mainly reflect female preschool educators’ experiences.

The expert group covered a wider age range. The oldest expert was included because she is widely recognized in China as a scholar and practitioner in preschool music education and remained actively engaged in frontline teaching, university teacher training, and professional development for early childhood music educators at the time of the study.

### Data collection and ethics

Data were collected through one-to-one semi-structured interviews. Data were collected through one-to-one semi-structured interviews. A semi-structured interview format was selected because it allowed participants to describe their professional understandings and practice-based experiences in their own terms, while enabling the interviewer to probe and clarify meanings related to a construct that remains under-specified in preschool music education [34,35]. Accordingly, the interview guide was purpose-built to elicit practitioners’ and experts’ understandings of music subject key competencies for preschool children aged 3–6. Guided by a pedagogical content knowledge perspective, the guide focused on how participants understood the educational value of preschool music learning, what competencies they considered important for children aged 3–6, how these competencies could be cultivated through teaching strategies and activity design, how children with different learning needs could be supported, and how music learning could be evaluated in everyday practice.

To strengthen clarity and contextual fit, the draft guide underwent expert review (three preschool education experts and two preschool music education experts) and was refined following pilot interviews with five frontline teachers from two kindergartens. A shared semi-structured interview guide was used across participant groups, with role-adapted follow-up questions when necessary.

Data collection commenced on 18 April 2024 with participant recruitment and interview scheduling and was completed on 13 June 2024. Each interview lasted approximately 30–60 minutes (kindergarten-based practitioners: 30–45 minutes, because their responses tended to focus on concrete classroom practices and daily teaching experiences; experts: 45–60 minutes, because they often provided more extended reflections on curriculum policy, music education theory, competency construction, and teacher development, which required additional probing and follow-up questions). Prior to participation, all participants received written study information and provided written informed consent; interviews were audio-recorded with permission. This study received ethical clearance from the Human Research Ethics Committee of Universiti Putra Malaysia (Jawatankuasa Etika Universiti Untuk Penyelidikan Melibatkan Manusia, JKEUPM), under approval reference JKEUPM-2022–938. During transcription, all materials were de-identified. Audio files, transcripts, and analytic notes were stored under access-controlled conditions and were used solely for research purposes.

### Inclusivity in global research

This study was conducted in the Chinese preschool education context and involved kindergarten-based practitioners and experts in preschool education and music education. The research design, participant recruitment, informed consent procedures, and interpretation of findings were developed with attention to the local educational, cultural, and ethical context. Additional information regarding the ethical, cultural, and scientific considerations specific to inclusivity in global research is included in the Supporting Information (S1 File).

### Data analysis

This study adopted an inductive qualitative analysis informed by grounded theory coding procedures. Here, “inductive” means that codes, categories, and higher-order dimensions were developed from the interview data rather than imposed in advance from an existing theoretical or curricular framework. This approach was appropriate because the study aimed to explore practitioners’ and experts’ understandings of preschool children’s music subject key competencies and, on this basis, develop a practice-grounded framework rather than test a pre-existing model. At the same time, these understandings were interpreted within the curricular and professional contexts in which participants worked, where existing competency concepts may already have shaped how preschool music learning was understood and described. Grounded theory coding procedures supported this process through iterative comparison and progressive category development, enabling detailed accounts to be developed into more abstract competency categories [36,37].

Data were managed in NVivo 12 Plus. All transcripts were read repeatedly before initial codes were generated from descriptions of children’s music-learning experiences, observable behaviours, and participants’ practice-based reasoning. Related codes were compared and grouped into preliminary categories, which were refined through repeated review and research-team discussion.

The 71 initial indicator codes were refined into 24 secondary indicators according to the meaning of each code, the behaviour or capacity represented, and its level of conceptual abstraction. Codes representing the same underlying competency were merged, whereas those reflecting different developmental functions were retained separately. Where category boundaries were unclear, the researchers returned to the original interview excerpts and compared them across participants. The 24 secondary indicators were then compared at a higher conceptual level and grouped into dimensions according to their shared competency focus and conceptual distinctiveness. Coding frequency was not used to determine the higher-order structure. Through this iterative process, the 24 secondary indicators were organized into the final seven-dimensional framework.

### Adequacy of themes and trustworthiness

Theme adequacy was considered throughout the analysis by examining whether the developing categories adequately captured the range of meanings represented across the dataset.

The first author conducted the interviews and led the initial coding and category development. The second and third authors participated in reviewing and refining the emerging categories and higher-order dimensions. The first author maintained reflexive notes during data collection and analysis to document emerging interpretations, prior assumptions, and uncertainties encountered during the analytic process. These notes provided a record of the researcher’s evolving understanding of the data. Decisions concerning category meanings and boundaries were based on repeated comparison with the original interview data and discussion among the research team.

Following Lincoln and Guba’s [38] framework, rigor was ensured through four criteria.

1. Credibility was supported through iterative discussion, supervisory review, and inclusion of representative quotations to maintain alignment between data and interpretation.
2. Transferability was enhanced by sampling across diverse geographic and institutional contexts and providing detailed descriptions of the research setting.
3. Dependability was ensured through systematic record-keeping of major analytic decisions and NVivo-based data management.
4. Confirmability was supported through reflexive notes, documentation of analytic decisions, and repeated checking of interpretations against the original interview data.

### Dimension-based findings

The analysis identified seven dimensions of preschool children’s music subject key competencies: Music Aesthetic Perception, Artistic Expression, Musical Social Skills, Cultural Understanding, Autonomous Development, Imagination and Creativity, and Physical Movement. These dimensions formed the higher-order structure through which participants’ accounts of preschool music learning were organized. They are understood as complementary and interrelated aspects of music learning rather than as sequential developmental levels.

The relationships between the seven dimensions and their secondary indicators are presented in Fig 1. Table 2 provides representative interview evidence for the indicators, while S1 Table further specifies the conceptual scope and boundaries of each dimension.

**Table 2:** A concise evidence matrix of indicators for preschool children’s music subject key competencies.

Overarching dimension	Second-level indicator	Initial indicator code	Representative Participant Excerpt	Dominant Sources (T/M/E)
Music Aesthetic Perception	1.1 Musical Perception	1.1.1 Sensing and Experiencing the Sounds of Nature or Life	E_LDN_: I guide children to close their eyes and quietly listen to the variety of sounds inside and outside the classroom—such as birdsong, wind, raindrops, footsteps—and then encourage them to imitate these sounds with their voices, respond through body movement, or use drawing and short verbal descriptions to express what they have heard and felt.	T, M, E
Artistic Expression	2.1 Integrated Arts Performance	2.1.2 Learning Performance Excerpts From Different Musical Genres (Such As Opera, Musical Theatre, Nursery Rhymes, Symphonic Music, etc.)	T_SMY_: I encourage children to try out different performance styles, for instance short fragments of opera, simple children’s songs and small percussion ensembles.	T, M, E
Musical Social Skills	3.2 Collaboration	3.2.2 Able to Proactively Adjust and Support the Team Throughout the Cooperation Process	T_LJJ_: During chorus or small-group performances, I notice that some children pay close attention to how their peers are doing. For example, when a classmate falls behind the beat, they will spontaneously slow down their own movements; when they notice a mistake, they gently remind or help correct it so that the overall performance can continue smoothly. This kind of awareness of others is an important sign of team spirit in music activities.	T, M, E
Cultural Understanding	4.3 Music and Other Subjects	4.3.1 Music with Mathematical Skills 4.3.2 Music with Visual-Spatial Skills 4.3.3 Language Skills 4.3.4 Music with Visual Arts	E_GNN_: From the perspective of early childhood music education, integrating music with other subjects not only enhances the enjoyment of teaching but also improves its effectiveness and depth. For example, when combining mathematics and music, musical rhythm and meter can support preschool children in understanding fundamental mathematical concepts such as counting, sequencing, and patterns. Through playful activities like drumming or clapping along to different rhythms, children naturally acquire mathematical skills while having fun. The integration of language and music is also highly effective—music and songs serve as powerful tools for language instruction and vocabulary expansion. Through singing and rhyming, preschool children gain a deeper understanding of language structure, while also improving their phonological awareness and expressive abilities. Music can also serve as an entry point to explore scientific concepts, such as sound vibrations and transmission. Through hands-on musical activities—like making simple instruments or investigating how different objects produce sounds—preschool children learn how sound is generated and altered, developing both their scientific curiosity and understanding. The fusion of visual-spatial learning with music is equally important. In my teaching practice, I often guide children to combine music with spatial imagination and movement planning, such as following the rhythm to arrange shapes or move along specific spatial pathways. This approach not only develops their spatial perception but also deepens their grasp of musical rhythm and structure. Moreover, integrating visual arts with music greatly enriches the expressive forms of early childhood education. We often organize interdisciplinary activities that combine music with painting or crafts, encouraging preschool children to express the imagery they hear in music through drawing, or to create artworks related to musical themes. Such multisensory and multimedia teaching models greatly stimulate children’s artistic interests and creativity. Social learning and music are closely connected as well. Since music is deeply embedded in social and cultural contexts, exploring music from various cultures helps preschool children broaden their worldview and develop greater respect for cultural diversity. This interdisciplinary teaching approach not only makes music education more diverse and enriching, but also supports children’s holistic development across multiple domains, enhancing their overall learning capacity and interest. In this way, music serves as a bridge that links knowledge from various subjects, making early childhood education more comprehensive and engaging.	T, M, E
Autonomous Development	5.1 Curiosity	5.1.3 Observational Ability	T_LXW_: I believe that preschool children have remarkable observational skills. They often notice details that adults tend to overlook, pointing out phenomena that teachers may not have anticipated. Such keen observation not only benefits their artistic learning but also fosters cognitive development in their everyday lives.	T, M, E
Imagination and Creativity	6.1 Imagination	6.1.1 Situational Association	T_YL_: Once, when I played a particularly lively piece, several children said it sounded like little rabbits jumping in the grass. Others said it felt like flowers blooming in the park in spring. Their imagination is incredibly rich; they frequently come up with ideas that adults would never think of.	T, M, E
Physical Movement	7.2 Reaction Ability	7.2.3 Movement Transition	T_WJ_: In some music games, children have to change movements constantly—for example, from clapping to stamping, then to spinning around. Some children can switch between these actions smoothly, without getting stuck, and their movements link together very naturally.	T, M, E

T: Preschool teacher

M: Manager

E: Expert

**Fig 1:**
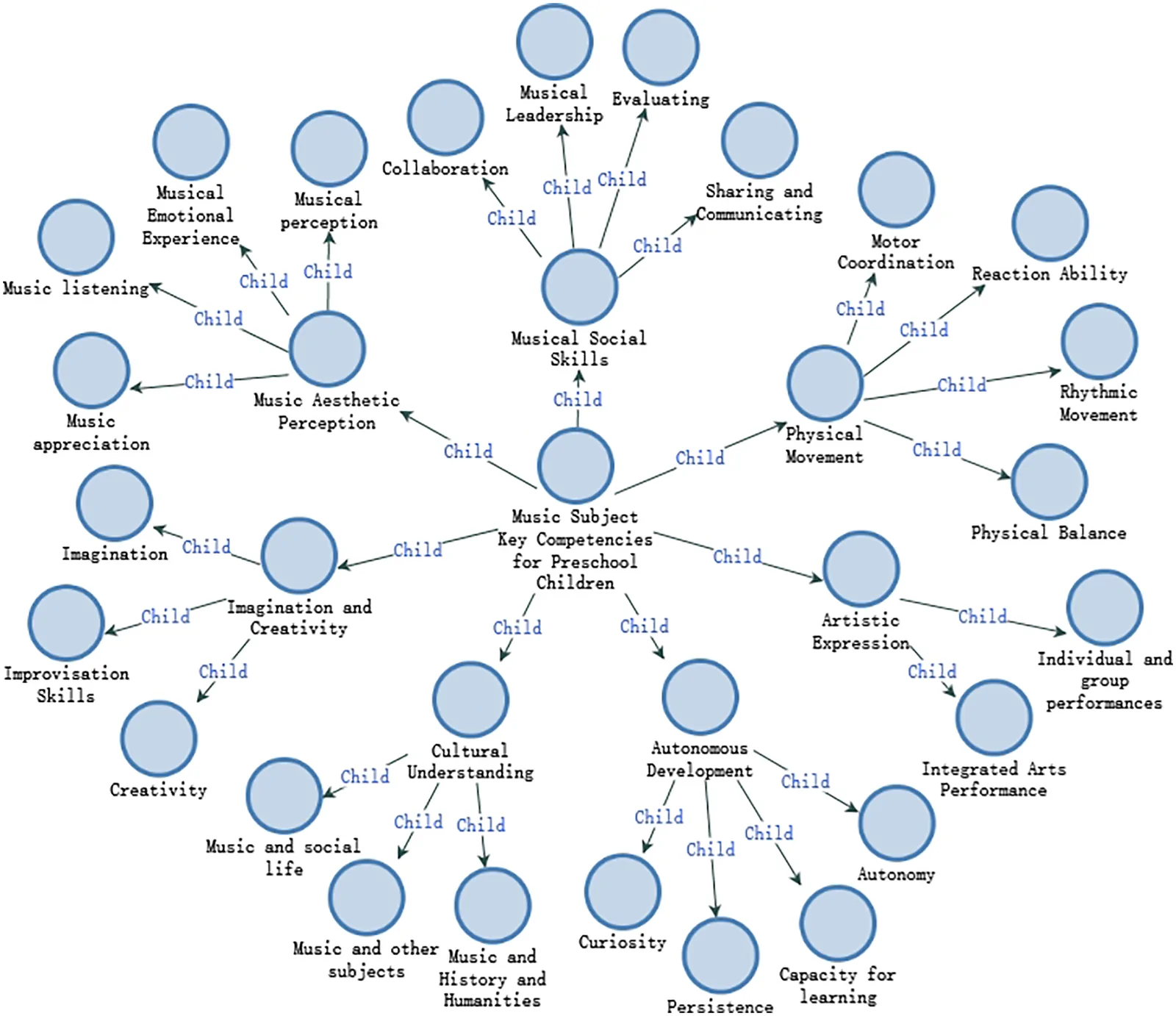
Network diagram of the seven-dimensional structure and secondary indicators of music subject key competencies for preschool children.

### Dimension–indicator evidence and distribution

To make the qualitative evidence transparent and easy to trace, the interview materials were organized into an evidence matrix in the form of “dimension—second-level indicator—representative short quotation—role coverage.” As shown in Table 2, each initial indicator code is accompanied by a representative excerpt that illustrates how participants described observable learning behaviours or pedagogical situations relevant to that competency element. In this sense, Table 2 presents the findings thematically by linking each competency dimension and indicator to representative interview evidence.

Role coverage is reported across three participant categories—teachers (T), managers (M), and experts (E)—to highlight both shared emphases and role-specific accents in how the same competency was articulated. Although teachers and managers were combined as kindergarten-based practitioners in Table 1, they were distinguished here to show whether each indicator was supported by classroom-level, management-level, and expert-level perspectives.

The excerpts in Table 2 are presented as evidence anchors rather than exhaustive data displays. Full operational definitions for each dimension, derived from aggregated coding and cross-case comparisons, are provided in S1 Table to ensure clear conceptual boundaries and consistent interpretive use across the manuscript.

### Qualitative synthesis and indicator distribution

Table 3 provides supplementary information on the distribution of indicators across the dataset. The number of sources indicates how widely an indicator appeared across participants, while the number of references reflects the amount of coded material associated with it. These counts were used descriptively and did not determine the importance or structure of the seven dimensions.

**Table 3:** Number of sources and number of references for indicators of preschool children’s music subject key competencies.

Second-level indicator	Initial indicator code	Number of Sources	Number of References
1.1 Musical Perception	1.1.1 Sensing and Experiencing the Sounds of Nature or Life	22	17
	1.1.2 Perceiving Basic Musical Elements		18
	1.1.3 Perceiving Musical Structure		13
1.2 Music Appreciation	1.2.1 Understanding Music Through Art Forms	16	15
	1.2.2 Understanding Artistic Characteristics of Musical Genres		15
	1.2.3 Understanding Different Music Styles		15
1.3 Music Listening	1.3.1 Identifying Voices and Instruments	11	11
	1.3.2 Identifying Musical Genres and Forms		10
1.4 Musical Emotional Experience	1.4.1 Feeling Emotions Expressed by Music	24	24
	1.4.2 Experiencing Musical Imagery		23
	1.4.3 Emotional Responses to Music Interaction		19
2.1 Integrated Arts Performance	2.1.1 Participation in Various Comprehensive Art Performance Activities (Rhythmic Activities, Music Games, Preschool Children’s Singing and Dancing Performances, etc.)	22	20
	2.1.2 Learning Performance Excerpts From Different Musical Genres (Such as Opera, Musical Theatre, Nursery Rhymes, Symphonic Music, etc.)		11
2.2 Individual and Group Performances	2.2.1 Solo and Choral Singing	23	21
	2.2.2 Solo and Ensemble Playing		22
	2.2.3 Sight-Singing Music Scores		7
3.1 Sharing and Communicating	3.1.1 Able to Actively Express Personal Feelings and Ideas During Music Activities	28	9
	3.1.2 Able to Listen Attentively and Respond Positively to Peers’ Musical Experiences and Expressions		6
	3.1.3 Able to Engage in Emotional Communication with Peers During Musical Interactions		5
3.2 Collaboration	3.2.1 Able to Work Together with Peers to Complete Shared Musical Activities	23	15
	3.2.2 Able to Proactively Adjust and Support the Team Throughout the Cooperation Process		4
	3.2.3 Able to Share the Results of Cooperation with Peers and Experience the Joy of Team Success		5
3.3 Evaluating	3.3.1 Evaluating Self or Peers	8	5
	3.3.2 Evaluating Music		3
3.4 Musical Leadership	3.4.1 Able to Take on Leadership Roles Such as Lead Singer or Conductor During Music Activities	11	6
	3.4.2 Able to Motivate Peers to Actively Participate in Music Activities		3
	3.4.3 Able to Organize and Coordinate the Team to Complete Musical Tasks		2
4.1 Music and History and Humanities	4.1.1 Perception and Understanding of the Stylistic Features and Cultural Contexts of Music Works From Various Countries and Ethnic Groups	12	26
	4.1.2 Fostering National Pride		11
	4.1.3 Environmental Awareness Through Music		5
4.2 Music and Social Life	4.2.1 Exploring the Connections Between Music and Nature or Social Contexts	4	2
	4.2.2 Exploring the Connections Between Music and Regional, Linguistic, or Cultural Practices		3
4.3 Music and Other Subjects	4.3.1 Music with Mathematical Skills	24	7
	4.3.2 Music with Visual-Spatial Skills		9
	4.3.3 Language Skills		23
	4.3.4 Music with Visual Arts		13
5.1 Curiosity Behavior	5.1.1 Questioning	8	4
	5.1.2 Investigative Behavior		7
	5.1.3 Observational Ability		5
5.2 Persistence	5.2.1 Concentration	13	4
	5.2.2 Patience		2
	5.2.3 Self-Control		12
5.3 Autonomy	5.3.1 Understanding	27	18
	5.3.2 Active Participation		11
	5.3.3 Confidence		13
	5.3.4 Problem-Solving Skills		5
	5.3.5 Awareness of Rules		3
	5.3.6 Cognitive Competence		4
5.4 Capacity for Learning	5.4.1 Memory	20	11
	5.4.2 Imitation		8
	5.4.3 Reflection		6
	5.4.4 Music Interest		12
6.1 Imagination	6.1.1 Situational Association	26	12
	6.1.2 Imagery Reproduction		8
	6.1.3 Story Expansion		6
6.2 Creativity	6.2.1 Fluency	27	15
	6.2.2 Flexibility		6
	6.2.3 Originality		6
6.3 Improvisation Skills	6.3.1 Immediate Response	6	1
	6.3.2 On-site Creation		2
	6.3.3 Theme Relevance		3
7.1 Motor Coordination	7.1.1 Fine Coordination	25	13
	7.1.2 Gross Coordination		12
7.2 Reaction Ability	7.2.1 Reaction Speed	16	7
	7.2.2 Movement Accuracy		5
	7.2.3 Movement Transition		4
7.3 Physical Balance	7.3.1 Static Balance	11	4
	7.3.2 Dynamic Balance		7
7.4 Rhythmic Movement	7.4.1 Rhythm Imitation	17	9
	7.4.2 Rhythmic Expression		4
	7.4.3 Rhythm Synchronization		4

Number of sources = the number of distinct interviews in which a given code appears; number of references = how many times that code is cited across the full dataset

Within Music Aesthetic Perception, participants placed particular emphasis on the emotional dimension of children’s music learning. Teachers and experts repeatedly described preschool music learning as extending beyond cognition and skill acquisition to include the experience and expression of emotion. They noted that, through music activities, children could experience and communicate a range of feelings and gradually develop greater awareness of their own emotional responses and ways of regulating them. Musical Emotional Experience appeared in 24 interviews and was coded 66 times, indicating that this aspect was not only widely represented across participants but also elaborated in considerable depth within their accounts.

Participants also commonly regarded Musical Perception as a foundational component of children’s music learning. They described it as providing important groundwork for subsequent musical performance, creative engagement, and aesthetic development. Some participants further connected musical perception with broader perceptual development and cognitive understanding. Musical Perception appeared in 22 interviews and was coded 48 times, indicating that this understanding was represented across a substantial proportion of the dataset. These accounts are presented as participants’ interpretations rather than as direct evidence of cross-domain transfer.

In discussing Sharing and Communication and Collaboration, teachers and experts often referred to choral singing, cooperative movement, and music games as situations in which children had to listen to others, coordinate their actions, and work toward a common task. Their accounts suggested that these activities created opportunities for children to express ideas, negotiate roles, follow shared rules, and adjust their behaviour in response to peers. Several participants also associated such interactions with the gradual development of emotional and behavioural self-regulation. Sharing and Communication was identified in 18 interviews with 20 coded references, while Collaboration appeared in 23 interviews with 24 references, showing that both forms of social interaction were recurrent across the dataset.

When discussing Music and Other Subjects, interviewees frequently described music as being connected with physical education, science, visual arts, and language learning. They saw these connections as a way of extending children’s experiences and offering different routes for making sense of what they encountered in music activities. Some accounts also linked musical participation with movement, spatial awareness, and the recognition of patterns or simple logical relationships. This indicator appeared in 24 interviews and generated 52 coded references, showing that cross-subject connections recurred across the dataset. These findings are interpreted as participants’ views of curriculum and pedagogy, rather than as evidence that music learning necessarily produces transfer to other domains.

Within Autonomous Development, autonomy was mainly described through children’s initiative and growing independence in music activities. Participants referred to children choosing whether and how to take part, sustaining their involvement, and making their own judgments during performance, improvisation, and group work. They also noted that children sometimes adjusted their actions in response to the demands of the activity rather than relying entirely on adult direction. Autonomy appeared in 27 interviews and was coded 54 times, showing that this aspect of development was widely represented across the participant groups.

Several indicators, including Evaluating Music, Musical Leadership, and Improvisation Skills, occurred less often in the interview data. Their lower coding frequencies were not taken to indicate lesser importance. Instead, these indicators were retained because they represented distinct aspects of participants’ accounts that were not adequately captured by the more frequently coded categories. Their presence therefore broadened the range of competencies represented in the framework, even though they were less extensively discussed across the interviews.

Within Artistic Expression, participants described children’s musical expression mainly through singing, instrumental playing, movement, and individual or group performance. Their accounts emphasized active participation in varied forms of performance rather than technical reproduction alone. For Imagination and Creativity, participants referred to children’s ability to form musical images, develop associations, vary familiar material, and respond spontaneously during musical activities. These accounts portrayed creativity as extending beyond imitation toward more flexible and self-generated expression. Physical Movement was reflected in coordination, reaction, balance, and rhythmic movement. Participants commonly described bodily responses as an integral part of children’s engagement with rhythm, musical change, and movement-based activities.

In Table 3, the number of sources reflects how broadly an indicator was represented across interviews, whereas the number of references reflects how extensively it was discussed in the coded material. These counts were used only to describe patterns in the dataset and were not treated as measures of the relative importance of individual competencies. Because the study used purposive sampling, participant groups were unequal in size, and coded excerpts were qualitative rather than independent quantitative observations, no inferential comparisons were made across teachers, managers, and experts.

## Discussion

### Contribution of this study: a practice-grounded framework

This study developed a preliminary, interview-derived seven-dimensional framework of music subject key competencies considered important for preschool children aged 3–6 years. The resulting framework therefore represents an inductive synthesis of how practitioners and experts conceptualize and organize competencies relevant to young children’s music learning, rather than a definitive or prescriptive specification of preschool music competencies.

Some overlap with existing curriculum concepts is expected, as participants’ accounts were formed within established curricular and professional contexts. The contribution of the present study therefore does not rest on claiming seven entirely new competency domains, but on specifying how these domains are organized and given meaning within preschool music learning.

Within this practice-grounded structure, the seven dimensions describe different but connected aspects of children’s engagement with music. Music Aesthetic Perception and Artistic Expression concern children’s direct engagement with musical experience through perceiving, feeling, performing, and expressing. Musical Social Skills and Cultural Understanding place this engagement within interpersonal and cultural contexts, while Autonomous Development and Imagination and Creativity capture the increasingly self-directed and generative qualities of children’s participation. Physical Movement reflects the embodied character of preschool music learning, through which children often perceive, respond to, and express music. These dimensions are analytically distinguishable but not independent: the same musical activity may involve several dimensions simultaneously. They also differ in the degree to which they are specific to music. Music Aesthetic Perception and Artistic Expression are directly rooted in musical perception and expression, whereas Musical Social Skills, Cultural Understanding, Autonomous Development, Imagination and Creativity, and Physical Movement overlap with capacities that may also develop in other educational contexts. Within the present framework, however, these broader capacities are included only when they are manifested through musical participation, musical materials, or music-related interaction and meaning-making; they are not treated as general developmental outcomes produced by music education. The framework therefore represents an integrated configuration of competencies rather than a hierarchy of discrete skills or developmental stages, while retaining a clear focus on how these competencies are expressed within preschool music learning.

Compared with existing curriculum frameworks that commonly identify broad processes such as perceiving, performing, creating, responding, and connecting, the present framework provides a preschool-specific account of how musical perception, expression, embodied action, social participation, autonomy, creativity, and cultural meaning are related in practice. In this sense, it moves beyond listing competency domains by clarifying how different forms of competence are organized and enacted within preschool musical participation.

On this basis, relevant early childhood music research provides further support for understanding how the dimensions may operate together within musical activity. Research on preschool musical interaction, early-years music-making, and music-and-movement pedagogy indicates that interaction, sound exploration, bodily movement, affective response, imagination, and creative expression are closely intertwined within musical activity [39–41]. From this perspective, preschool music provides a context in which multiple competencies may be expressed and connected through participation, rather than functioning as isolated developmental outcomes.

These findings can also be considered in relation to broader international and Chinese perspectives on music and arts education. The New Zealand Curriculum and the National Core Arts Standards conceptualize music learning through interrelated processes such as responding, performing, creating, and connecting [42,43], while Chinese scholarship emphasizes the developmental and practical character of music key competencies [44,45]. The present study extends this work by specifying, through practitioners’ and experts’ interview perspectives, how these competencies are organized and related within preschool music learning.

At the same time, the developmental relevance of the framework should not be interpreted as evidence of automatic cross-domain transfer. Transfer is conditional rather than automatic [46], and music-specific evidence also supports a cautious position. Sala and Gobet [47] questioned strong claims about broad cognitive and academic benefits of music training, while Mehr et al. [48] found no consistent evidence that brief preschool music enrichment improved non-musical cognitive outcomes. More recent reviews suggest that music training may be associated with specific executive function outcomes, such as inhibitory control, working memory, and cognitive flexibility, rather than unrestricted developmental transfer across all domains [49,50]. Accordingly, these findings should be interpreted as showing how multiple capacities can be brought together within children’s musical participation, rather than as evidence of transfer to non-musical domains.

### Preschool music learning revealed by the seven dimensions

The seven dimensions highlight three interrelated characteristics of preschool music learning: its experiential basis, its expressive and embodied forms, and its social and cultural situatedness. The significance of the seven dimensions lies not only in establishing a classificatory framework, but also in revealing how preschool music learning is assigned developmental value in authentic educational understanding.

### Musical perception and emotional experience: the experiential foundation of preschool music learning

Musical perception and emotional experience together position early music learning as an experiential process in which musical meaning is encountered through sensing, feeling, association, and response. From this perspective, music perception may provide an experiential foundation for subsequent musical expression, creativity, and aesthetic development.

This interpretation can be related to Piaget’s view of cognitive development, according to which children gradually construct cognitive structures through repeated interactions with their environment [51]. In preschool music activities, listening, rhythmic games, movement responses, and sound exploration provide children with concrete sensory experiences, enabling them to notice patterns, distinguish contrasts, and organize musical meaning. However, this study does not claim that music perception directly leads to cognitive transfer. Instead, it understands music perception as a practice-based indicator of children’s developing sensitivity to sound, patterns, contrast, and expressive meaning.

Emotional experience further extends this experiential account by emphasizing music as a context for perceiving, expressing, and making sense of affective experience. This interpretation is consistent with Gardner’s [52] view that musical intelligence is an important way for children to perceive, organize, and express experience. It also echoes Goleman’s [53] argument that emotional awareness and expression are important for social adaptation and psychological well-being. Therefore, music aesthetic perception and musical emotional experience should be regarded as foundational components of the music subject key competencies for preschool children. Their value lies in emphasizing that children’s music learning is not merely a form of technical training, but a gradual process that unfolds through perception, feeling, association, response, and expression.

### Artistic expression, creativity, and physical movement: visible forms of musical understanding

Artistic expression, creativity, and physical movement reflect developmentally appropriate ways in which young children interpret, transform, and communicate musical experience. Together, these dimensions highlight the expressive, imaginative, and embodied nature of preschool music learning.

This interpretation is consistent with Chen’s [54] empirical study on the use of musical approaches to support the development of creative thinking in 5–6-year-old children. Chen argued that, within the context of key competencies-oriented education and multiple intelligences theory, music activities can create meaningful conditions for the development of young children’s creative thinking. This perspective supports the understanding of the present study that imagination and creativity are not merely additional outcomes of preschool music education, but important ways through which children transform musical experiences into personal expression.

In addition, research on music education and motor creativity has shown that music activities can provide structured opportunities for children to explore movement, coordination, and improvisation [55]. Studies on embodied music learning have also emphasized that bodily movement helps children understand music and participate in creative expression [56]. Therefore, physical movement should not be regarded simply as an auxiliary teaching strategy. Rather, it can be understood as an important pathway through which children perceive, organize, and express musical meaning.

Together, these dimensions suggest that musical understanding in early childhood is expressed not only through what children reproduce, but also through how they embody, transform, and communicate musical meaning.

### Musical social skills, autonomous development, and cultural understanding: the contextual value of preschool music learning

Musical social skills place children’s musical participation within a shared interpersonal context. This interpretation is consistent with Vygotsky’s view that children’s development occurs through social interaction, shared activity, and cultural practice [57]. It also echoes recent research suggesting that music, rhythm, and movement activities may provide supportive environments for children’s social participation and self-regulation [20,22]. This study further defines musical social skills as a specific dimension of music subject key competencies for preschool children, rather than merely as a general outcome of social development.

Autonomous development similarly draws attention to the self-directed qualities of children’s musical participation, including their capacity to make choices, sustain engagement, and respond to challenges within music activities. This interpretation is consistent with research indicating that music activities may support the development of executive function and self-regulation in preschool children [50].

Cultural understanding, in turn, situates musical learning within children’s wider social and cultural worlds. This aligns with the sociocultural perspective that learning is embedded in cultural tools, social practices, and shared meanings [57]. Accordingly, this framing situates cultural and cross-curricular connections within preschool music practice rather than treating them as outcomes external to music learning.

Together, these dimensions show that preschool music learning is situated not only within individual musical experience, but also within interpersonal participation, self-directed engagement, and culturally meaningful activity.

### Implications for curriculum design and assessment: from broad aims to observable evidence

The findings of this study have important implications for the design and assessment of music curricula in preschool education. In preschool arts education, broad developmental aims can be difficult to translate into specific classroom objectives and observable evidence. The seven-dimensional framework provides a clearer basis for connecting broad competency aims with curriculum planning, classroom observation, and formative assessment in preschool music education, offering a structured reference for their interpretation and application in practice.

First, the framework helps clarify the goals of music education in preschool education. Rather than reducing curriculum goals to the mastery of particular songs, rhythms, or performance routines, it provides a more differentiated language for considering what children may perceive, express, create, negotiate, and embody through musical participation. In this sense, the framework provides a more specific language for discussing music learning in preschool education than general aims for arts education alone.

Second, the framework may offer preliminary guidance for process-oriented assessment. In many preschool education settings, music learning is more easily assessed through visible outcomes, such as whether children can sing a song, follow a rhythm, or complete a performance. However, such outcome-oriented assessment may overlook children’s listening ability, emotional responses, peer collaboration, creative variation, movement expression, and autonomous participation. This concern is consistent with Yu and Li’s [15] argument that assessment in music education should move beyond narrow judgments of outcomes and pay greater attention to musical experience, learning processes, and children’s development. By identifying observable behavioral descriptions within each dimension, the framework provides a foundation for future research on classroom observation and formative assessment in preschool music education.

Third, the framework can inform the design of developmentally appropriate music activities. Used as reference points rather than fixed teaching targets, the dimensions may help teachers consider whether music activities create sufficiently varied opportunities for children to listen, explore, express, interact, make choices, move, and connect musical experiences with wider cultural contexts. Therefore, the framework should be regarded as an initial reference for future curriculum and assessment development, rather than as immediate evidence of classroom effectiveness.

Overall, the framework offers a structured starting point for translating broad competency aims into more concrete pedagogical and assessment practices. Further research is still needed to translate this framework into concrete teaching examples, observation tools, and assessment rubrics, and to examine its applicability across different preschool education contexts.

## Implications

This study provides implications for preschool music curriculum development, teacher practice, and assessment. The seven-dimensional framework offers a more specific language for describing preschool children’s music subject key competencies and may help shift attention beyond song mastery, rhythmic accuracy, and performance display toward how perception, emotional response, expression, creativity, peer interaction, autonomy, cultural understanding, and physical movement are manifested within children’s participation in music activities. The dimensions and indicators identified in this study may serve as preliminary reference points for translating broad arts aims into more concrete teaching and assessment practices. Further empirical research is needed to examine the framework across different preschool contexts and to develop teaching examples, assessment rubrics, and observation tools based on it.

## Limitations

The conclusions of this study were drawn within a specific research context and design, and therefore should be interpreted with certain boundaries. First, the interview sample was mainly drawn from Henan Province. Although it included frontline teachers, managers, and experts in preschool education and music education, the regional and cultural backgrounds of the participants were relatively concentrated. In addition, the participating kindergartens were primarily model kindergartens with relatively stable institutional histories, sufficient music-related resources, and regular music activities or performances. Such samples provided rich practical experience for the exploratory construction of the framework, but they may also overrepresent institutions with stronger resources and more established music education practices. Therefore, the seven-dimensional framework proposed in this study should not be regarded as a direct reflection of all preschool education institutions in China. Its applicability across different regions, cultural contexts, and types of kindergartens requires further examination. Moreover, all kindergarten practitioners involved in this study were female. Although this is consistent with the female-dominated composition of the preschool education workforce in China, it may also have shaped the practical perspectives reflected in the findings.

Second, the framework developed in this study was mainly constructed through qualitative interviews and inductive qualitative analysis. Its structural validity, developmental pathways, and practical effects have not yet been verified through large-scale surveys, structural equation modelling, longitudinal tracking, or classroom-based intervention studies. Future research could therefore validate the framework across a wider range of kindergarten types and regional contexts. Further efforts are also needed to develop age-appropriate assessment tools, classroom observation scales, and teaching cases. Cross-regional surveys, experimental studies, and action research could be used to examine the stability and relative weight of each dimension, as well as the relationships between these dimensions and preschool children’s music learning performance.

## Conclusion

Based on interview data collected within the context of Chinese preschool education, this study examined the conceptualization and structural articulation of music subject key competencies for preschool children aged 3–6. The study developed a seven-dimensional framework comprising music aesthetic perception, artistic expression, musical social skills, cultural understanding, autonomous development, imagination and creativity, and physical movement. Rather than treating the broader social, autonomous, creative, cultural, and motor capacities represented in the framework as music-specific outcomes in themselves, the framework identifies how these capacities are manifested within children’s participation in musical activities. These dimensions also overlap with emphases already present in preschool music curricula, indicating that the framework refines rather than replaces existing understandings.

The main contribution of this study lies in refining existing understandings of preschool music learning by organizing these capacities into a coherent, practice-grounded framework and clarifying their conceptual boundaries and interrelationships within musical participation. Developed from the accounts of kindergarten practitioners and experts, the framework offers a practice-based reference for understanding preschool children’s music subject key competencies and for future curriculum and assessment development. At the same time, the findings should be interpreted within the limits of the qualitative design and regional context. The study does not establish causal effects or demonstrate that music learning automatically transfers to other developmental domains. Future research should further examine the framework through classroom observation, cross-regional validation, scale development, and intervention studies.

## Supporting information

S1 File Inclusivity in global research checklist. (DOCX)

S1 Table Coding-based definitions of music subject key competencies for preschool children. (DOCX)
