# Supplementary material for: Constructing preschool children’s music subject key competencies in Henan province, China: A theoretical study based on interviews with kindergarten teachers and experts
Source: PLoS One. 2026 Nov 13;21(11):e0348052. doi: 10.1371/journal.pone.0348052 (PMC13766293; doi:10.1371/journal.pone.0348052)
Supplement: Supplementary file 1 [file pone.0348052.s001.docx]

Inclusivity in global research

PLOS’ policy on inclusivity in global research aims to improve transparency in the reporting of research performed outside of researchers’ own country or community and ensures that PLOS publications reporting global research adhere to high standards for research ethics and authorship. Authors of relevant research articles may be asked to complete the questionnaire below, which outlines ethical, cultural, and scientific considerations specific to inclusivity in global research. This questionnaire may be requested when researchers have travelled to a different country to conduct research, if research uses samples collected in another country, research with Indigenous populations or their lands, or if research is on cultural artefacts. Researchers travelling to another country solely to use laboratory equipment will not normally be required to complete the questionnaire. However, the questionnaire can be requested at the journal’s discretion for any submission – if you have been requested to complete this questionnaire by the PLOS journal you submitted to, please do so.

Please complete the questionnaire below and include this as a Supporting Information file with your manuscript. Note that if your paper is accepted for publication, this checklist will be published with your article in the supporting information files. Please ensure that you reference the checklist in the main body of your manuscript. We suggest adding a subsection ‘Inclusivity in global research’ to your Methods section and adding the following sentence: “Additional information regarding the ethical, cultural, and scientific considerations specific to inclusivity in global research is included in the Supporting Information (SX Checklist)”

The questions have been designed to be applicable to a wide range of study types, and there are subsections for both human subjects research and non-human subjects research. If any of the questions are not relevant to your research please mark them as “N/A” as appropriate.

**Ethical considerations, permits and authorship**

*This section is applicable to all research types.*

Provide details as to who granted permissions and/or consent for the study to take place in the Methods section of your manuscript. This should include the names of **all** ethics boards, governmental organizations, community leaders or other bodies that provided approval for the study. If individuals provided approval refer to these people by their role or title but do not list their name(s).

Reported on page number: 10

If there were any deviations from the study protocol after approval was obtained please provide details of these changes in the Methods section of your manuscript.
Did this study involve local collaborators that are residents of the country where the research was conducted or members of the community studied? If you do not have any authors from said communities, please provide an explanation for this below.

Reported on page number: No deviations from the approved study protocol occurred.

Yes. The research involved first author and second author who are resident in China and familiar with the Chinese preschool education context and the community studied. They contributed to study design, participant recruitment, contextual interpretation, data analysis, and manuscript preparation. All authors meet PLOS authorship criteria.

Everyone listed as an author should meet PLOS’ criteria for authorship and all individuals who meet these criteria should be included in the author byline, rather than the acknowledgements. For further information please see the journal’s Authorship Policy.

**Human subjects research (e.g. health research, medical research, cross-cultural psychology)**

Did you obtain written informed consent from a representative of the local community or region before the research took place? How did you establish who speaks for the community? Details of written informed consent obtained from study participants should be reported separately in the Methods section of your manuscript.

This study did not require consent from a community representative because it did not study a local or Indigenous community as a collective entity. The study involved individual adult participants, including kindergarten teachers, managers and preschool or music education experts. Site access was arranged through participating institutions where applicable, and written informed consent was obtained from every individual participant before the interviews.

How did members of the local community provide input on the aims of the research investigation, its methodology, and its anticipated outcome(s)?

Local educational input was incorporated during instrument development and data collection. The interview guide was reviewed by experts in preschool education and preschool music education and refined after pilot interviews with five frontline teachers from two kindergartens. Participants’ interview accounts also informed the construction and interpretation of the framework.

When engaging with the local community, how did you ensure that the informed consent documents and other materials could be understood by local stakeholders?

All participant-facing materials, including the study information sheet, informed consent form, and interview guide, were provided in Chinese and written in clear, non-technical language. Participants were adult education professionals. The researcher explained the study purpose, voluntary nature of participation, confidentiality arrangements, and right to withdraw before obtaining written consent.

Will the findings of the research be made available in an understandable format to stakeholders in the community where the study was conducted (e.g. via a presentation, summary report, copies of publications, etc.)? Please provide details of how this will be achieved.

Yes. The findings will be made available to interested participating kindergartens and stakeholders through a concise Chinese summary and/or copies of the publication after publication. Where appropriate, findings may also be shared through academic exchange, teacher training, or professional development activities related to preschool music education.

**Non-human subjects research using specimens/ animals collected as part of the study, or those housed in archival collections. Examples include archaeology, paleontology, botany and zoology.**

Did the permission you obtained from a local authority to perform the study include an agreement on access to outputs and benefit sharing? This may include procedures to enable fair distribution of the benefits and resources arising from the research performed. Please include any details of Prior Informed Consent and Benefit Sharing Agreements obtained. These may be required by field-specific regulations, for example the Convention on Biological Diversity (CBD) and the associated Nagoya Protocol.

N/A. This was human subjects interview research and did not involve specimens, animals, archival collections, field collections, or benefit-sharing agreements under the Convention on Biological Diversity or Nagoya Protocol.

If the material used in your study was imported, please A) provide the year it was imported and B) indicate whether permits were obtained to import/export the materials used, C) provide details of any permits obtained. If this information is not available, please indicate this.

N/A. No materials, specimens, animals, or archival objects were imported or exported for this study.

If you used archival specimens, please state how the material used in your study was acquired by the institute it is held in and provide details of any permits obtained for the original excavations/ sample collection. If this information is not available, please indicate this.

N/A. The study did not use archival specimens or archival collections.

How was the potential cultural significance of the materials collected in your study to local communities considered in your research design? Were Indigenous peoples and/or local researchers and institutions involved with archaeological excavations / collection of specimens? If so, please provide a description of their involvement.

N/A. The study did not collect cultural artefacts, archaeological materials, specimens, or materials from Indigenous peoples or their lands. It collected interview data from adult education professionals. Cultural context was considered through local expertise, Chinese-language materials, ethical approval, informed consent, and contextual interpretation of the findings.

If your manuscript includes photographs of human remains please indicate whether authors obtained permission from descendants or affiliated cultural communities to do so.

N/A. The manuscript does not include photographs of human remains.
