# Supplementary material for: Constructing preschool children’s music subject key competencies in Henan province, China: A theoretical study based on interviews with kindergarten teachers and experts
Source: PLoS One. 2026 Nov 13;21(11):e0348052. doi: 10.1371/journal.pone.0348052 (PMC13766293; doi:10.1371/journal.pone.0348052)
Supplement: Supplementary file 2 [file pone.0348052.s002.docx]

**S4 Table**.** Coding-based definitions of **music subject key competencies** for preschool children.**

| **Overarching dimension** | **Definition** |
| --- | --- |
| **Music Aesthetic Perception** | Music aesthetic perception **refers to the ability of preschool children to actively engage in music appreciation through the integrated perception and differentiation of elements such as pitch, melody, rhythm, and timbre.** Through attentive listening and musical analysis, preschool children gradually develop a sensitivity to musical beauty and form individualized aesthetic judgments. This process also enables them to perceive and express a wide range of emotions conveyed in music, such as joy, calmness, or excitement. The development of this ability not only contributes to the overall enhancement of their musical literacy but also lays a critical foundation for their artistic creativity and aesthetic education. |
| **Artistic Expression** | Artistic expression refers to the ability of preschool children to engage in creative expression and presentation through various artistic forms during music education activities. This dimension highlights both their expressiveness and level of participation in individual and group performances. Under the guidance of teachers, preschool children actively take part in singing, instrumental playing, rhythmic movement, and other musical activities, demonstrating their understanding of musical content and emotional responses. In integrated arts settings, they are able to harmonize sound, movement, and situational context, thereby enhancing the coherence and emotional impact of their artistic expression. Individual performances foster self-confidence and creativity, while group performances promote a sense of collaboration and collective identity. The development of artistic performance not only enriches the aesthetic experiences of preschool children but also lays a solid foundation for their holistic development and social adaptability. |
| **Musical Social Skills** | Musical social skills refers to the social qualities preschool children demonstrate during music activities. It encompasses their ability to actively share and communicate musical experiences with peers, engage collaboratively in ensemble playing and group performances, and exhibit a strong sense of teamwork and adherence to rules. Through interactive processes, preschool children are encouraged to evaluate and provide feedback on both their own musical performance and that of others, fostering mutual understanding and self-improvement. In addition, preschool children demonstrate a certain degree of musical leadership by taking the initiative to organize, coordinate, and guide their peers during group activities, thereby facilitating the smooth completion of collective tasks. This dimension highlights the cultivation of social interaction and collaboration within music activities, fostering the comprehensive development of preschool children’s social competence, sense of responsibility, and leadership awareness. |
| **Cultural Understanding** | **Cultural understanding** emphasizes fostering preschool children's deep awareness of diverse cultures and societies through music education. This dimension includes the appreciation and recognition of traditional Chinese culture and patriotic values. It encompasses not only the exploration of various musical genres and their historical development but also an understanding of how music reflects distinct cultural contexts and value systems. Through guided experiences, preschool children are encouraged to engage with and respect their national heritage, thereby enhancing cultural confidence and a sense of national identity.  Moreover, cultural understanding involves the meaningful integration of music with other subjects. While engaging in music-related activities, preschool children are introduced to interdisciplinary connections—linking music with language, literature, visual arts, physical education, and science. These cross-disciplinary experiences allow children to perceive and interpret the cultural dimensions of music from multiple perspectives. As a result, their cultural awareness is broadened, their interdisciplinary competencies are strengthened, and they develop open-minded, inclusive, and confident cultural attitudes. |
| **Autonomous Development** | **Autonomous development** refers to the capacity of preschool children to demonstrate self-direction, self-regulation, and active engagement during music activities. This dimension highlights the importance of allowing preschool children to independently choose from various types of music activities based on their personal interests and developmental needs. Within these activities, they are encouraged to express and explore their ideas proactively.  When encountering challenges or difficulties, preschool children are expected to exhibit sustained curiosity and a strong desire to explore. They are willing to try new approaches and persist in completing tasks, reflecting a high level of perseverance and self-discipline. Through these experiences, children gradually cultivate essential abilities such as self-management, intrinsic motivation, and autonomous learning.  Such development not only enhances their musical literacy but also lays a solid foundation for positive, proactive learning habits, independent thinking, and lifelong learning—traits essential for their future academic and personal growth. |
| **Imagination and Creativity** | Imagination and creativity refer to the rich imaginative capacity, innovative mindset, and improvisational expression demonstrated by preschool children during musical activities. Preschool children are able to envision and construct unique musical scenarios through musical imagination and creativity, actively exploring novel forms of expression in the processes of creation and performance. Their improvisational abilities are reflected in the flexible application of musical knowledge to engage in spontaneous playing, singing, or rhythmic movement. This dimension fosters independent thinking, innovative practice, and confident self-expression, representing a high-level competency within the music subject key competencies for preschool children. |
| **Physical Movement** | Physical movement refers to the ability of preschool children to interpret and express musical rhythm, structure, and emotion through bodily actions such as rhythmic movement, dance, and rhythmic imitation. This dimension emphasizes not only coordination, bodily control, and spatial awareness but also balance, reaction speed, and synchronization between movement and music. Within diverse and engaging musical activities, preschool children are able to seamlessly integrate music with physical expression, using dance and performance to convey their inner emotions. Furthermore, participation in group activities fosters teamwork and social interaction. Through the integration of music and bodily movement, children’s physical fitness and motor skills are effectively enhanced. Simultaneously, their capacity for emotional expression, creativity, and overall music subject key competencies for preschool children are nurtured, promoting the harmonious development of health and music education. |
